# Supplementary material for: No evidence for associations between brood size, gut microbiome diversity and survival in great tit (Parus major) nestlings
Source: Anim Microbiome. 2023 Mar 22;5:19. doi: 10.1186/s42523-023-00241-z (PMC10031902; doi:10.1186/s42523-023-00241-z)
Supplement: Supplementary file 1 — Additional file 1: Brood size before and after manipulation: brood sizes between treatment groups were tested with a linear model to see if the differences were statistically significant. [file 42523_2023_241_MOESM1_ESM.docx]

# **Supplementary file 10.** The gut microbiome alpha diversity (Shannon Diversity Index and Chao1 Richness) and short-term survival.


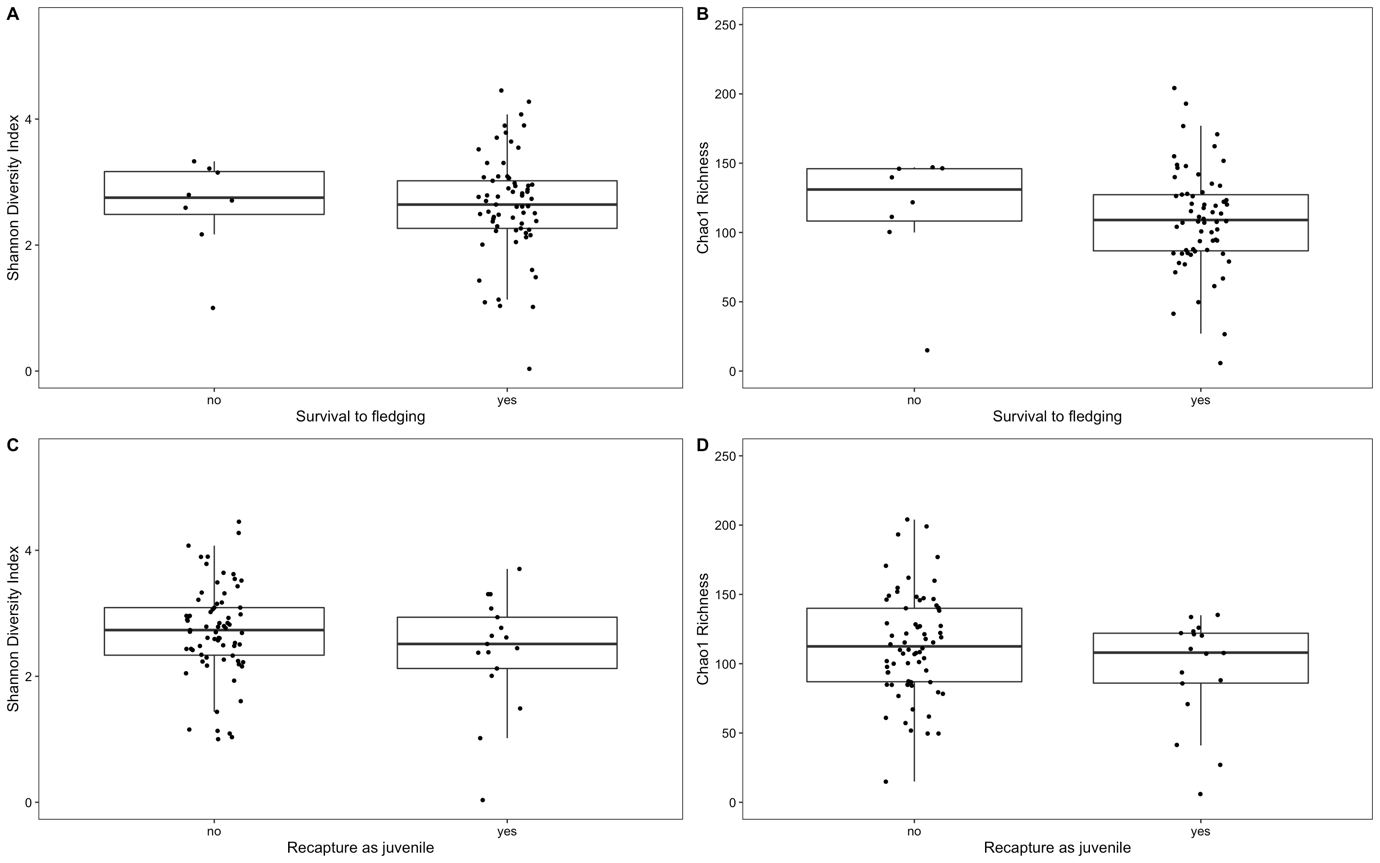


In survival to fledging (A: Shannon Diversity Index; B: Chao1 Richness) 65 nestlings fledged successfully and 8 nestlings were dead. 15 nestlings had no fledging record, so these were excluded from the analysis. In recapture as juvenile (C: Shannon Diversity Index; D: Chao1 Richness) 19 out of 92 (with data on microbiome diversity) were captured. The black dots represent each observation within a treatment group. The whiskers represent 95 % confidence intervals.
