# Supplementary material for: No evidence for associations between brood size, gut microbiome diversity and survival in great tit (Parus major) nestlings
Source: Anim Microbiome. 2023 Mar 22;5:19. doi: 10.1186/s42523-023-00241-z (PMC10031902; doi:10.1186/s42523-023-00241-z)
Supplement: Supplementary file 2 — Additional file 2: Rarefaction curves for the unrarefied dataset. Species (ASVs) plateaued at about 5000 reads which was used as the rarefying depth. [file 42523_2023_241_MOESM2_ESM.docx]

# **Supplementary file 11.** Generalized linear model to measure the association between alpha diversity (Shannon Diversity Index and Chao1 Richness) survival to fledging and apparent juvenile survival.


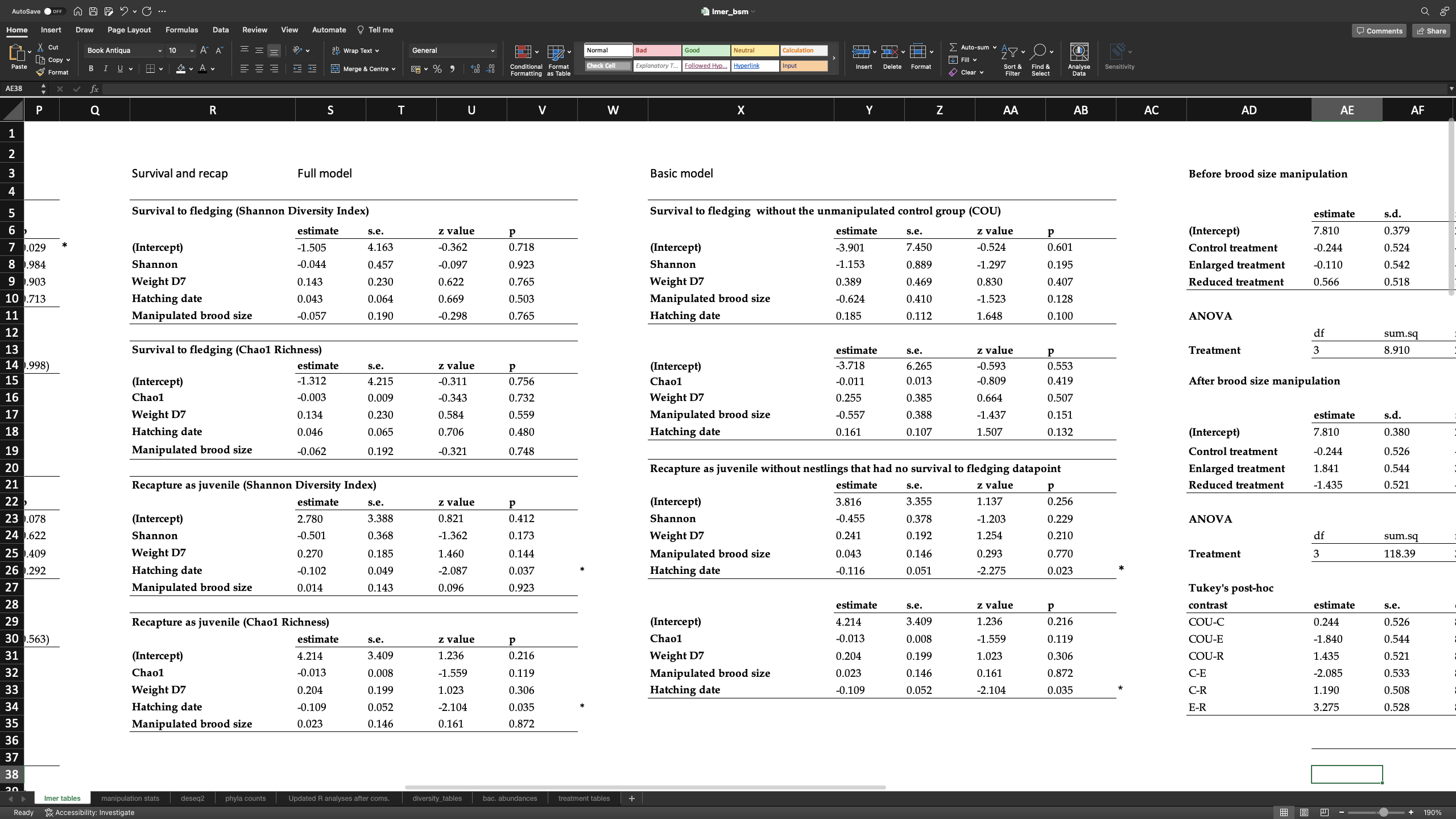


The survival to fledging model is a basic model including the control (C), enlarged (E), and reduced (R) treatment groups and individuals with no recorded fledging success removed. In the recapture as juvenile model, nestlings without a recorded fledging success (N=16) are removed from the data. To simplify both models, all random intercepts (nest of origin and nest of rearing) were removed as they contained 59 levels each and the model failed to converge when they were included.
