# Supplementary material for: No evidence for associations between brood size, gut microbiome diversity and survival in great tit (Parus major) nestlings
Source: Anim Microbiome. 2023 Mar 22;5:19. doi: 10.1186/s42523-023-00241-z (PMC10031902; doi:10.1186/s42523-023-00241-z)
Supplement: Supplementary file 3 — Additional file 3: Phylogenetic tree using the Newick-format. The tree describes the dissimilarity among the treatment groups. Each tip represents an individual sample, and each tip is colored and shaped based on treatment. Treatment groups are clustered using the UPGMA algorithm. [file 42523_2023_241_MOESM3_ESM.docx]

# **Supplementary file 12.** Ordination of the gut microbial communities.


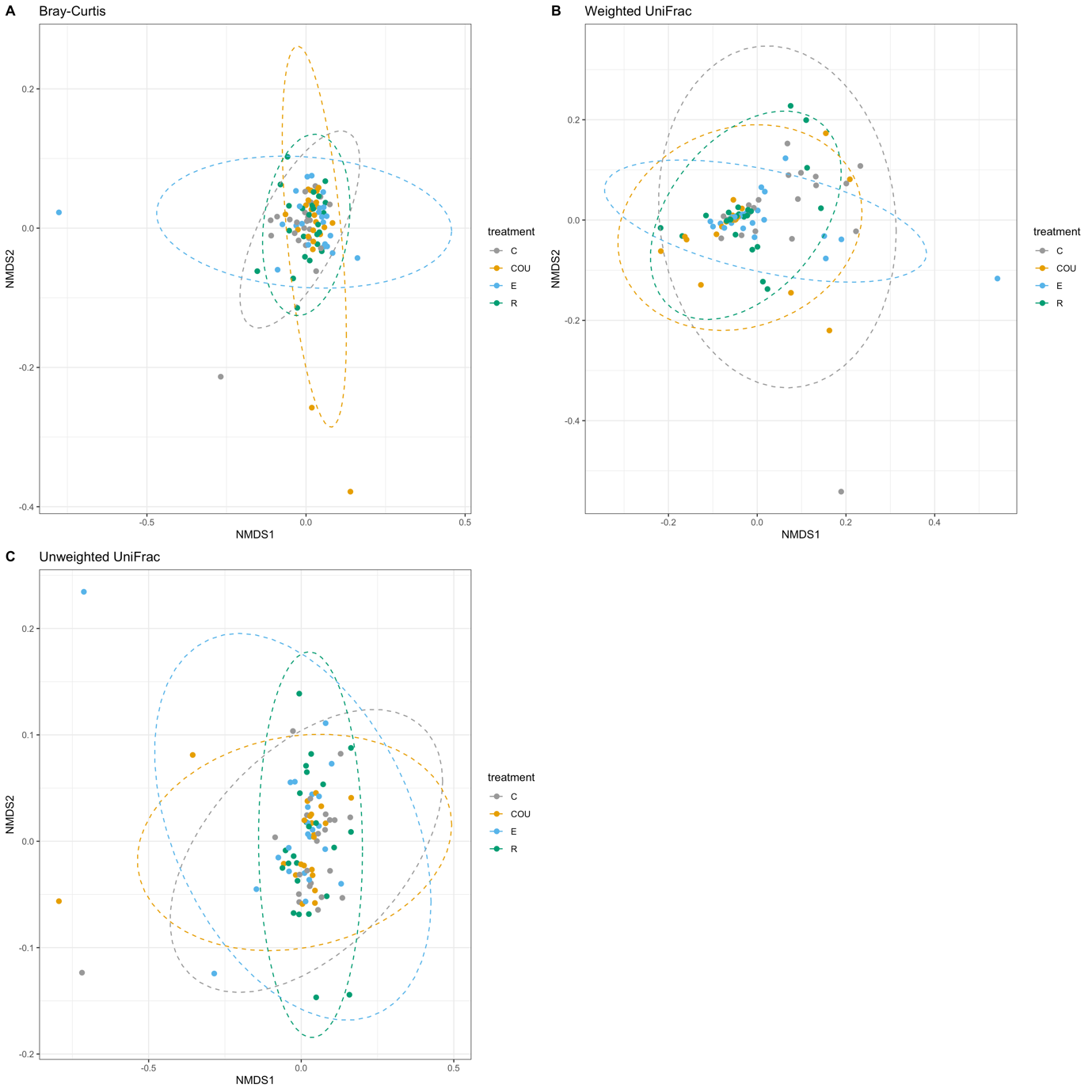


A) Weighted UniFrac, B) Unweighted UniFrac, and C) Bray-Curtis dissimilarity are displayed on NMDS ordinations. The color of the dots indicates which treatment, and the dashed ellipses represent 95 % confidence intervals.
