# Supplementary material for: No evidence for associations between brood size, gut microbiome diversity and survival in great tit (Parus major) nestlings
Source: Anim Microbiome. 2023 Mar 22;5:19. doi: 10.1186/s42523-023-00241-z (PMC10031902; doi:10.1186/s42523-023-00241-z)

# Supplementary file 13. Differential analysis of abundance (DESeq2) to assess the ASV abundance between the treatment groups


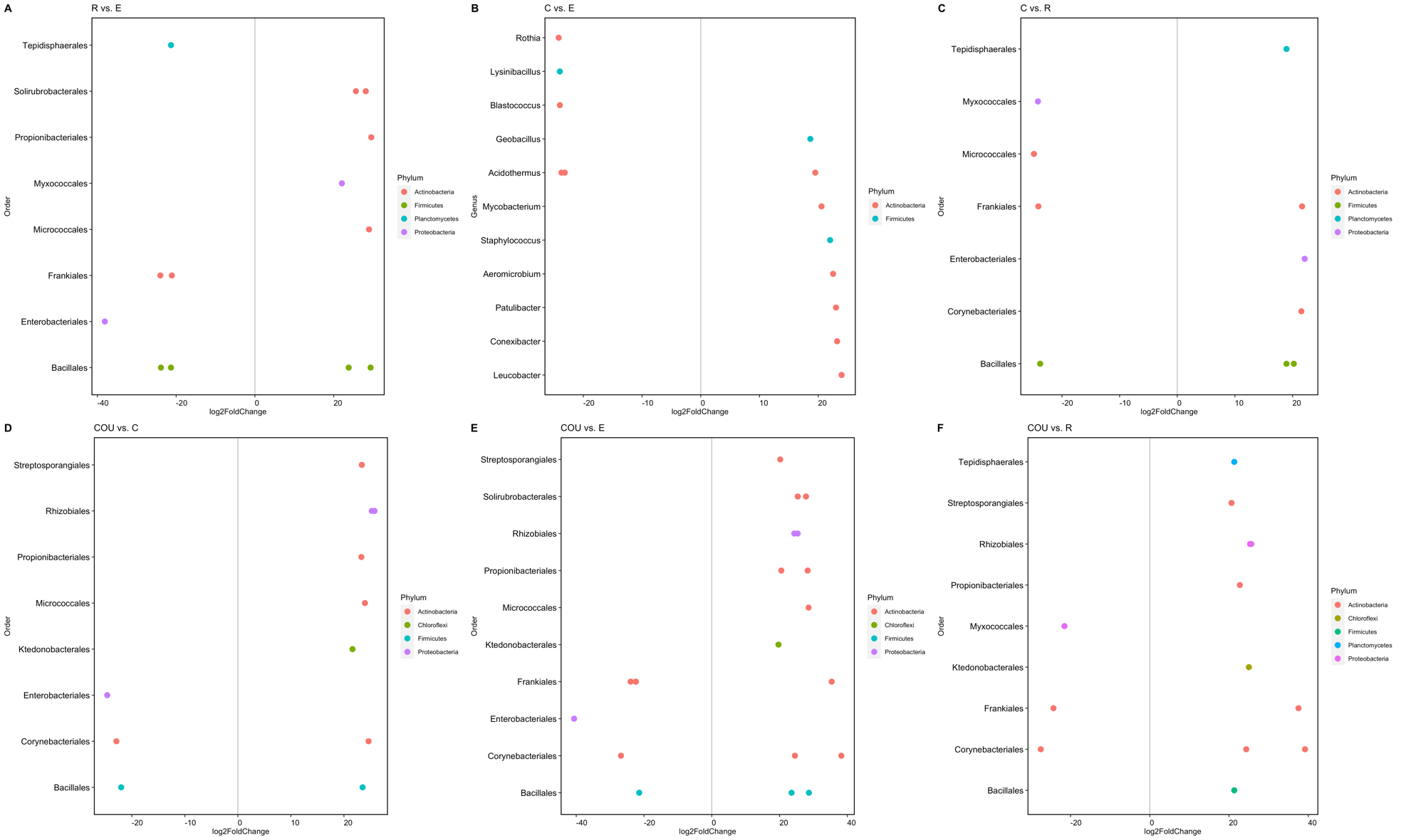

Supplement: Supplementary file 4 — Additional file 4: (A) Linear mixed effects model for gut microbiome diversity (Shannon Diversity Index and Chao1 Richness) and brood size manipulation treatment. (B) Linear mixed effects model for GM diversity (Shannon Diversity Index and Chao1 Richness) and manipulated brood size. [file 42523_2023_241_MOESM4_ESM.docx]
