# Supplementary material for: No evidence for associations between brood size, gut microbiome diversity and survival in great tit (Parus major) nestlings
Source: Anim Microbiome. 2023 Mar 22;5:19. doi: 10.1186/s42523-023-00241-z (PMC10031902; doi:10.1186/s42523-023-00241-z)
Supplement: Supplementary file 5 — Additional file 5: A linear mixed effects model investigating the effects of brood size manipulation on nestling body mass on day 7 and day 14 post-hatch. [file 42523_2023_241_MOESM5_ESM.docx]

Supplementary file 1. Brood size before and after manipulation. Brood sizes between treatment groups were tested with a linear model to see if the differences were statistically significant.

**
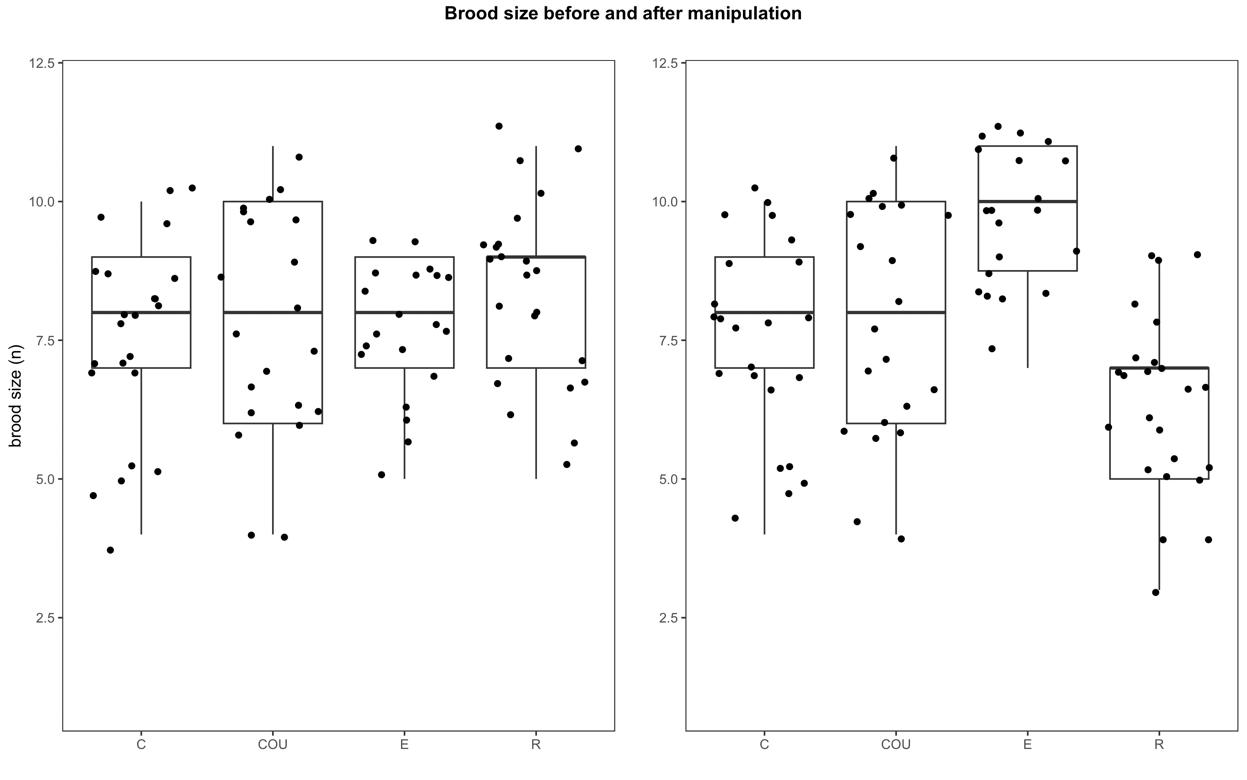
**

**
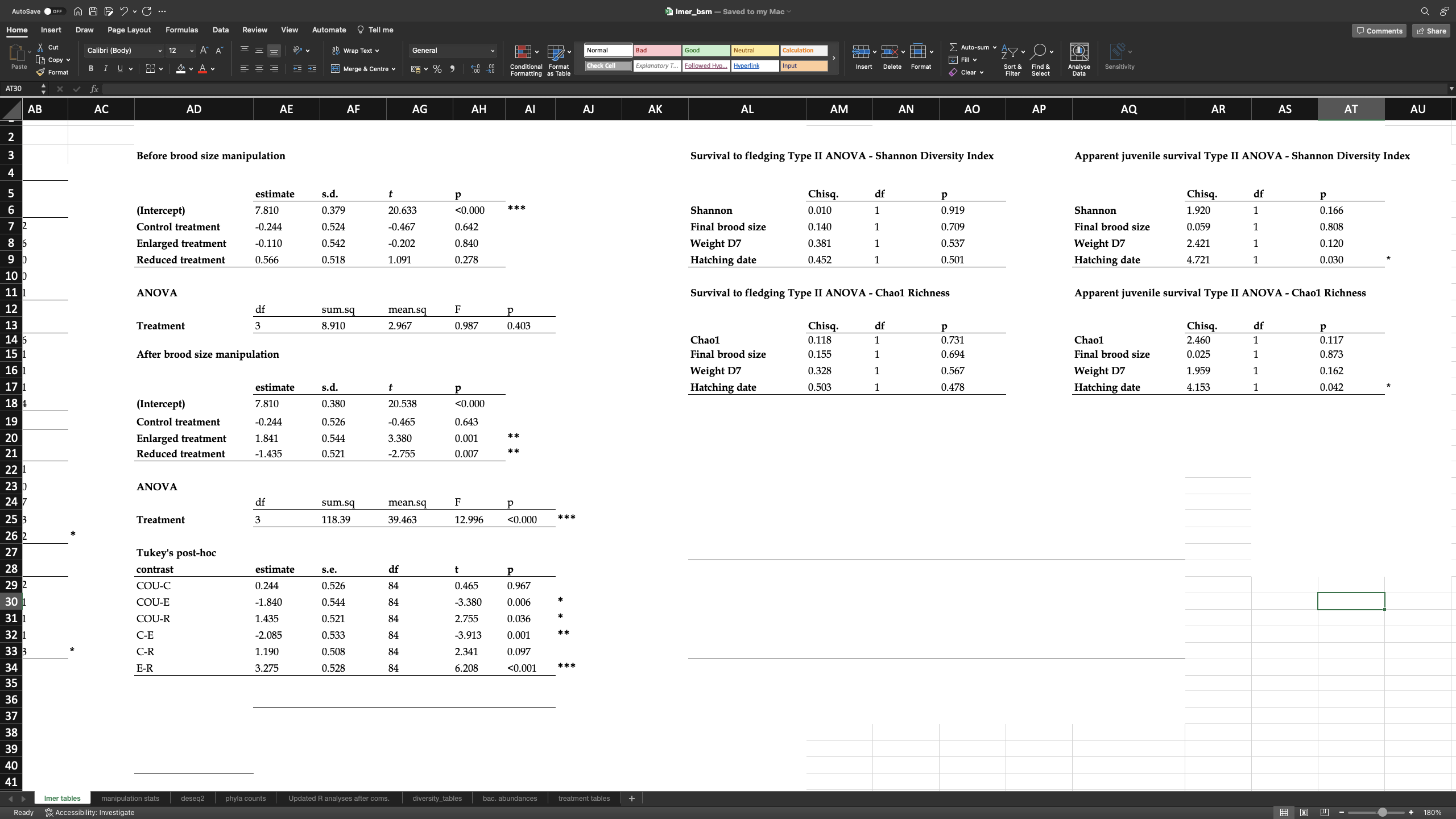
**
