# Supplementary material for: No evidence for associations between brood size, gut microbiome diversity and survival in great tit (Parus major) nestlings
Source: Anim Microbiome. 2023 Mar 22;5:19. doi: 10.1186/s42523-023-00241-z (PMC10031902; doi:10.1186/s42523-023-00241-z)
Supplement: Supplementary file 6 — Additional file 6: A linear mixed effects model investigating the effects of manipulated brood size on nestling body mass on day 7 and day 14 post-hatch. [file 42523_2023_241_MOESM6_ESM.docx]

Supplementary file 2. Rarefaction curves for the unrarefied dataset. Species (Amplicon Sequence Variants, ASVs) plateaued at about 5000 reads which was used as the rarefying depth.

**
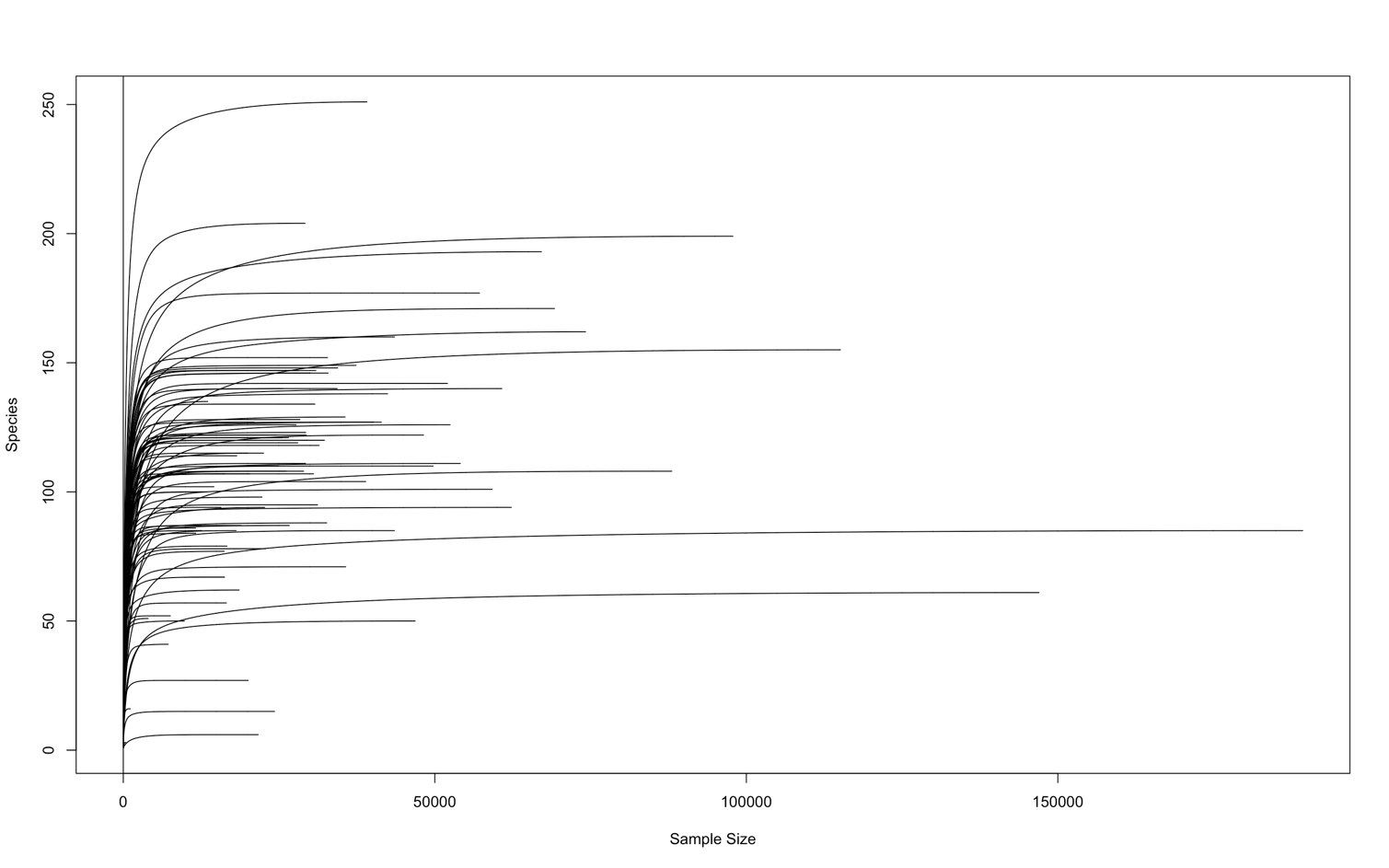
**
