# Supplementary material for: No evidence for associations between brood size, gut microbiome diversity and survival in great tit (Parus major) nestlings
Source: Anim Microbiome. 2023 Mar 22;5:19. doi: 10.1186/s42523-023-00241-z (PMC10031902; doi:10.1186/s42523-023-00241-z)

Supplementary file 3. Phylogenetic tree using the Newick-format. The tree describes the dissimilarity among the treatment groups. Each tip represents an individual sample, and each tip is colored and shaped based on treatment. Treatment groups are clustered using the UPGMA algorithm.


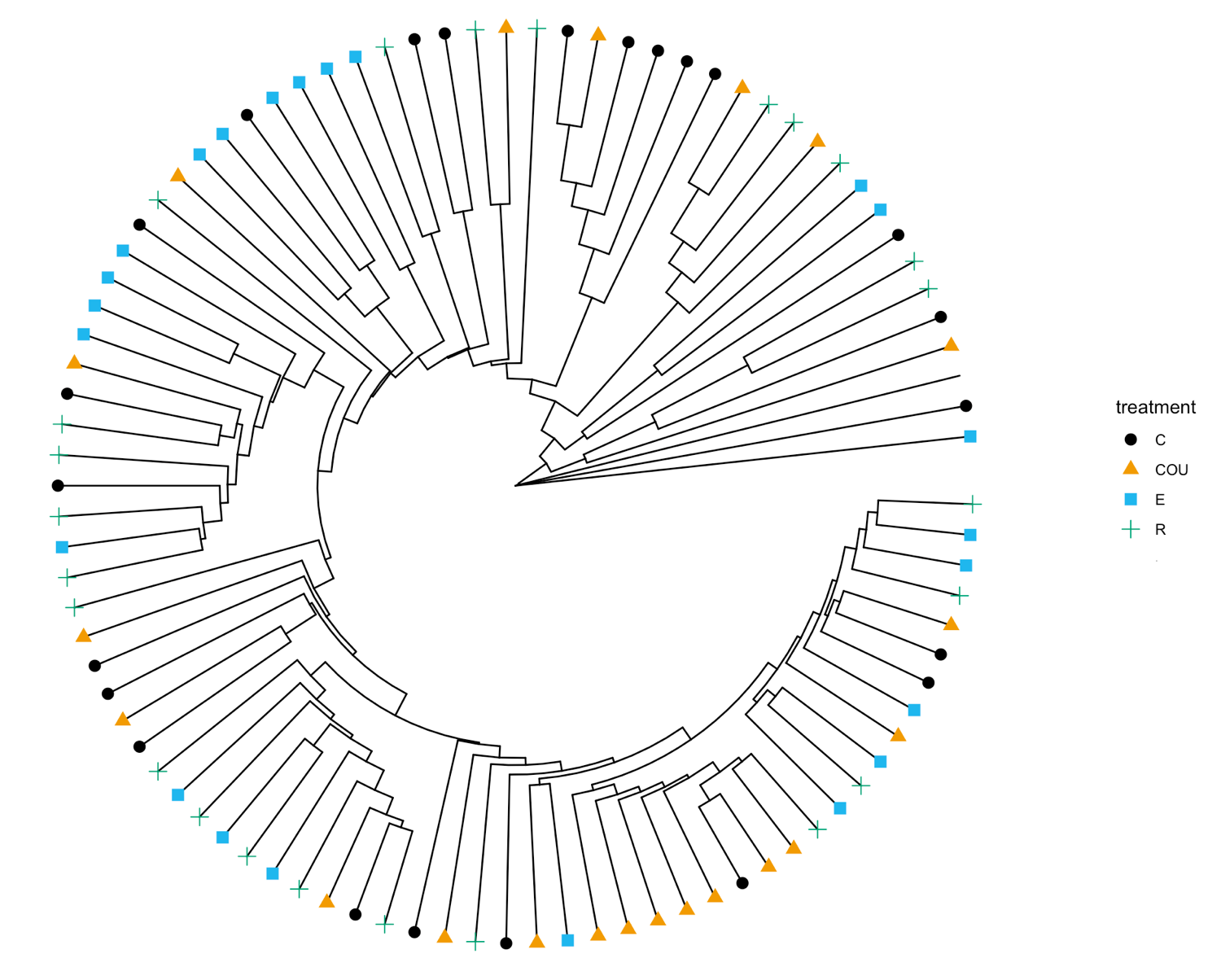

Supplement: Supplementary file 7 — Additional file 7: A linear mixed effects model investigating the associations between alpha diversity (Shannon Diversity Index and Chao1 Richness) and brood size manipulation. [file 42523_2023_241_MOESM7_ESM.docx]
