# Supplementary figures and images for: No evidence for associations between brood size, gut microbiome diversity and survival in great tit (Parus major) nestlings
Source: Anim Microbiome. 2023 Mar 22;5:19. doi: 10.1186/s42523-023-00241-z (PMC10031902; doi:10.1186/s42523-023-00241-z)

# Supplemental information 4. **Likelihood ratio test for random effect significance**

**
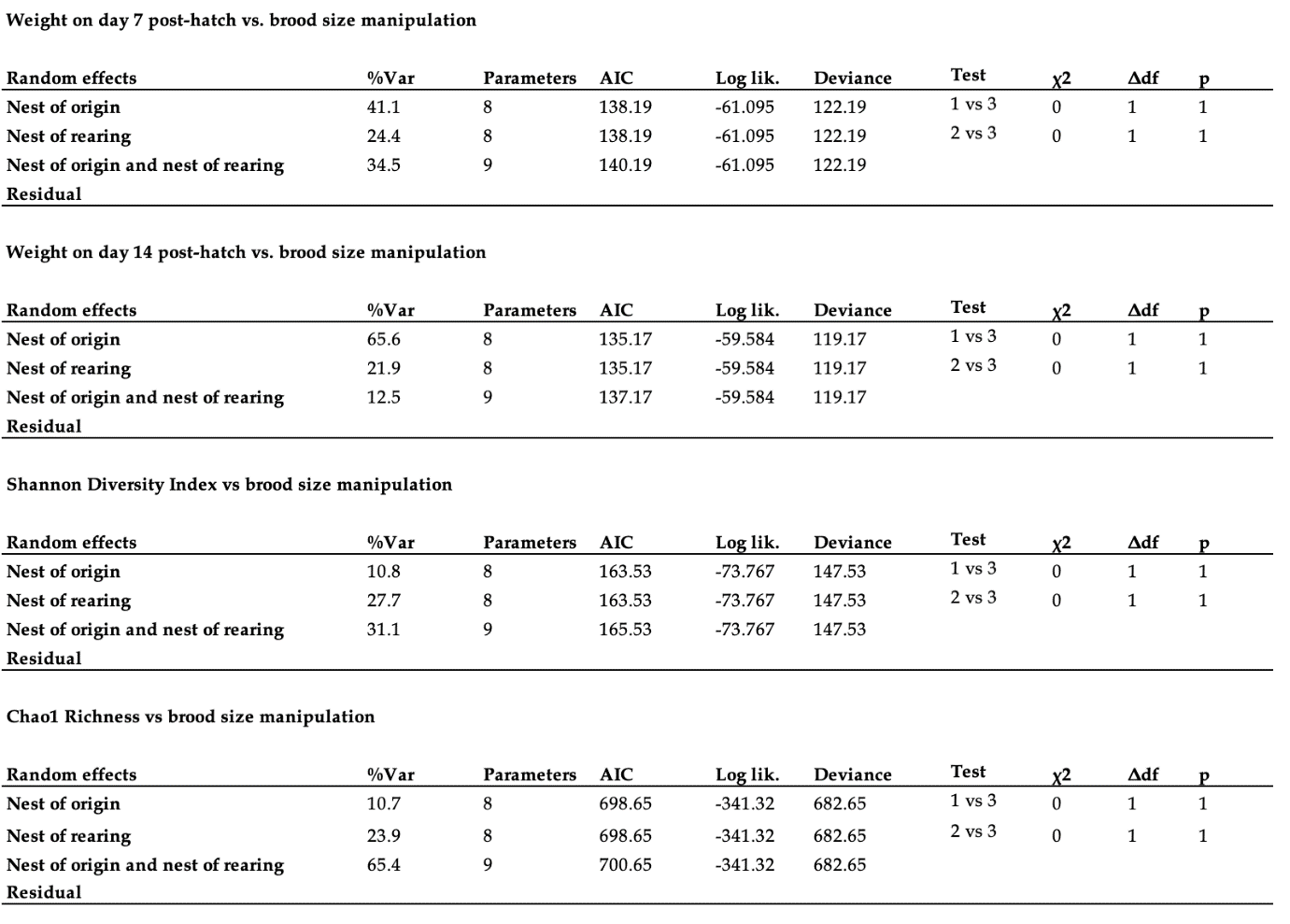
**

Supplement: Supplementary file 8 — Additional file 8: A linear mixed effects model investigating the association between alpha diversity (Shannon Diversity Index and Chao1 Richness) and manipulated brood size. [file 42523_2023_241_MOESM8_ESM.docx]
