# Supplementary material for: No evidence for associations between brood size, gut microbiome diversity and survival in great tit (Parus major) nestlings
Source: Anim Microbiome. 2023 Mar 22;5:19. doi: 10.1186/s42523-023-00241-z (PMC10031902; doi:10.1186/s42523-023-00241-z)
Supplement: Supplementary file 9 — Additional file 9: A generalized linear model exploration into alpha diversity’s (Shannon Diversity Index and Chao1 Richness) association with short-term (survival to fledging) and mid-term (apparent juvenile) survival. [file 42523_2023_241_MOESM9_ESM.docx]

# **Supplementary file 5.** A linear mixed effects model investigating the effects of brood size manipulation on nestling body mass on day 7 and day 14 post-hatch.

**
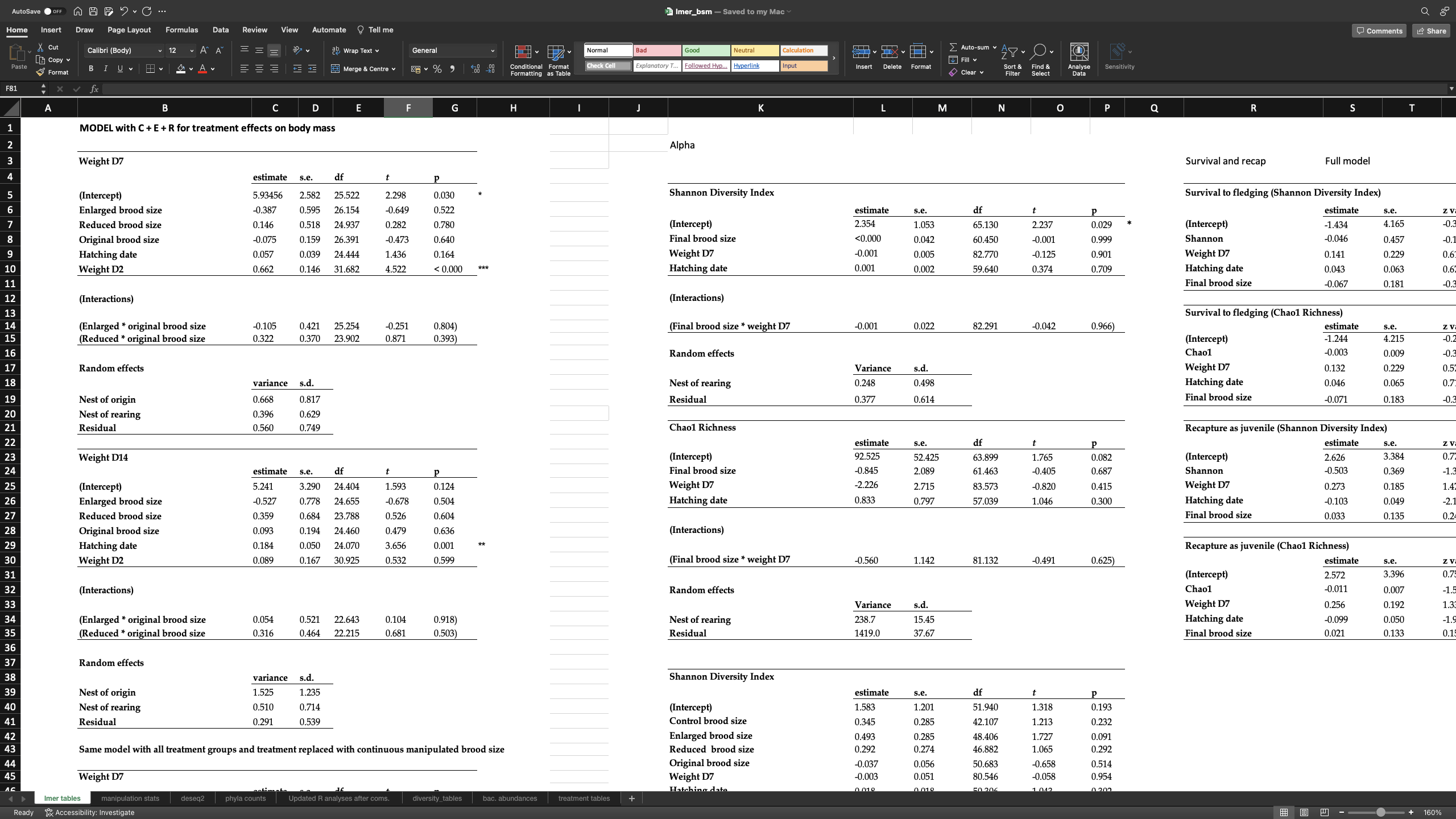
**

This basic model includes control (C), enlarged (E), and reduced (R) groups. Interactions between manipulated brood size and original brood size were removed from final models as there was no significant interaction and are shown in the table below. Nest of origin and nest of rearing were included as random effects to control for the non-independency of samples.

## Supplemental information 5.1: Supplements for covariates and random effects

*The effects of brood size manipulation on nestling body mass*

Original brood size (ANOVA: F1, 27.486=0.189, p=0.667, Table 1) and hatching date (ANOVA: F1, 25.813=1.712, p=0.202, Table S1) did not associate with nestling body mass on day 7 post-hatch. Weight on day 2 positively correlated with weight on day 7 post hatch (ANOVA: F1, 10.884=33.697, p=<0.000, Table S1). Variance explained by the nest of origin was estimated to be higher (σ2=0.698, s.d.=0.836) than by the nest of rearing (σ2=0.417, s.d.=0.646). Weight on day 2 post-hatch did not affect nestling body weight on day 14 post-hatch (ANOVA: F1, 10.600=0.032, p=0.861, Table S1). However, hatching date was positively correlated with nestling body weight on day 14 post-hatch (ANOVA: F1, 24.345=11.903, p=0.002, Table 1). There was no significant interaction between brood size manipulation and original brood size (ANOVA: F2, 23.223=0.219, p=0.805, Table S1). Variance explained by the nest of origin was lower (σ2=0.865, s.d.=0.930) than by the nest of rearing (σ2=0.996, s.d.=0.840).
