# Supplementary material for: No evidence for associations between brood size, gut microbiome diversity and survival in great tit (Parus major) nestlings
Source: Anim Microbiome. 2023 Mar 22;5:19. doi: 10.1186/s42523-023-00241-z (PMC10031902; doi:10.1186/s42523-023-00241-z)
Supplement: Supplementary file 10 — Additional file 10: The gut microbiome alpha diversity (Shannon Diversity Index and Chao1 Richness) and short-term survival. [file 42523_2023_241_MOESM10_ESM.docx]

# **Supplementary file 6.** A linear mixed effects model investigating the effects of final brood size on nestling body mass on day 7 and day 14 post-hatch.

**
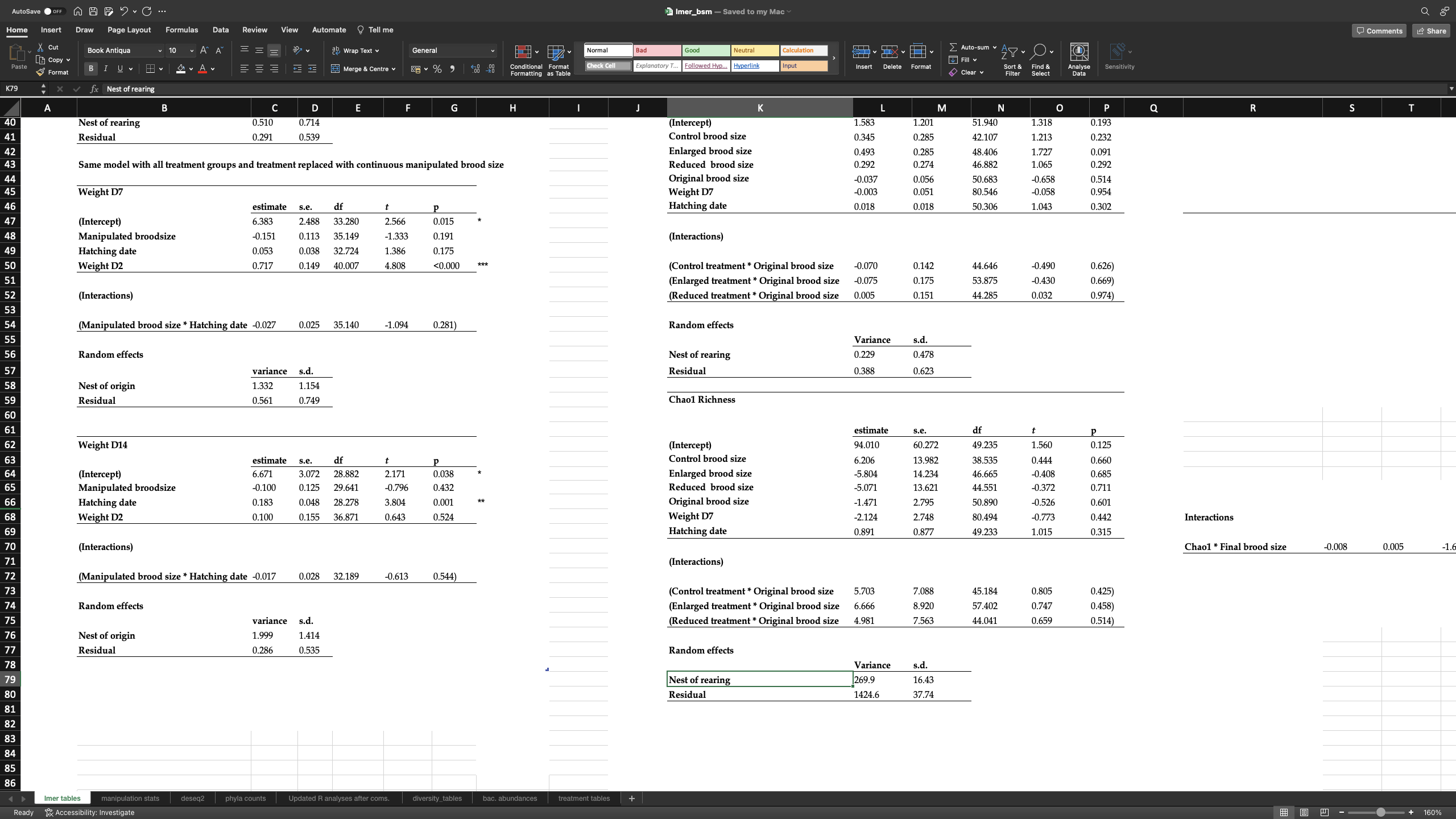
**

The analysis includes all treatment groups i.e., the full model: control (C), unmanipulated control (COU), enlarged (E), and reduced (R). Nest of origin was included as a random effect to control for the non-independency of samples.
