# Supplementary material for: No evidence for associations between brood size, gut microbiome diversity and survival in great tit (Parus major) nestlings
Source: Anim Microbiome. 2023 Mar 22;5:19. doi: 10.1186/s42523-023-00241-z (PMC10031902; doi:10.1186/s42523-023-00241-z)
Supplement: Supplementary file 11 — Additional file 11: Generalized linear model to measure the association between alpha diversity (Shannon Diversity Index and Chao1 Richness) survival to fledging and apparent juvenile survival. [file 42523_2023_241_MOESM11_ESM.docx]

# **Supplementary file 7.** A linear mixed effects model investigating the associations between alpha diversity (Shannon Diversity Index and Chao1 Richness) and brood size manipulation.

**
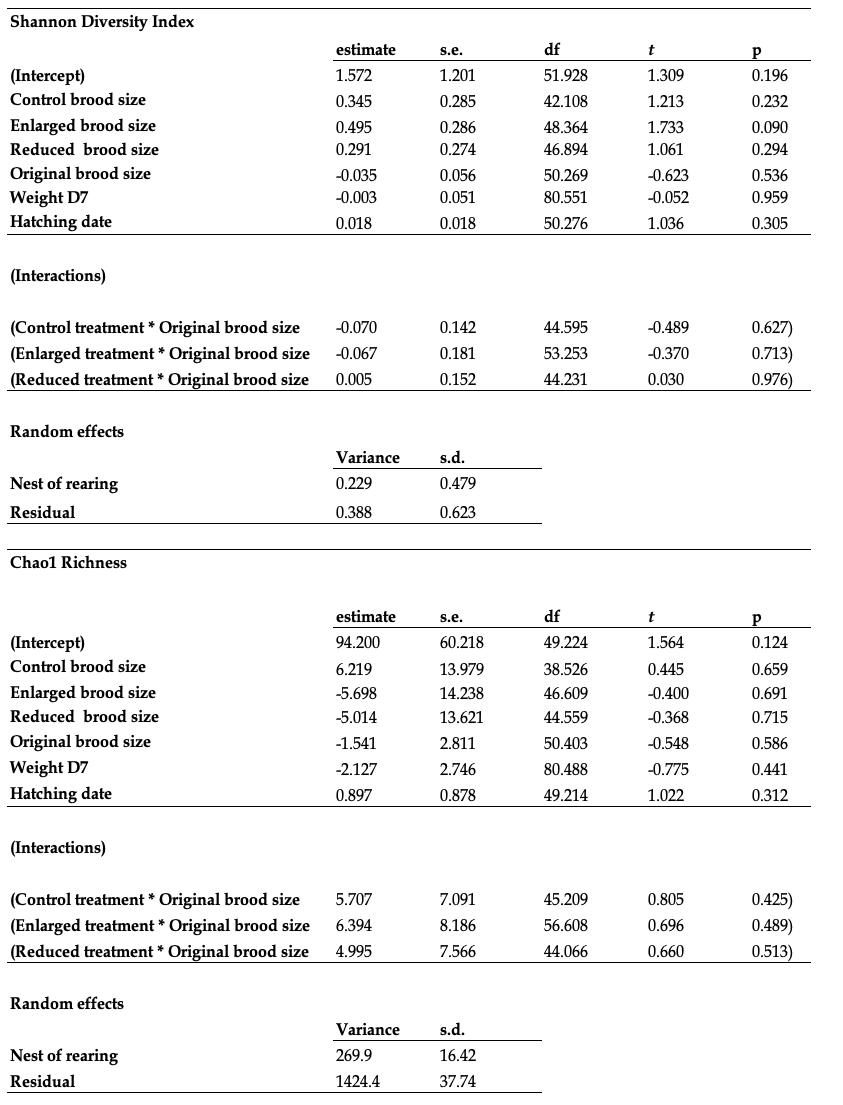
**

The model includes all four treatment groups i.e., the full model: control (C), unmanipulated control (COU), enlarged (E), and reduced (R). Interactions between brood size manipulation and original brood size were removed as there was no significant interaction and are shown in the table below. Nest of rearing was included as a random effect to control for the non-independency of samples.
