# Supplementary material for: No evidence for associations between brood size, gut microbiome diversity and survival in great tit (Parus major) nestlings
Source: Anim Microbiome. 2023 Mar 22;5:19. doi: 10.1186/s42523-023-00241-z (PMC10031902; doi:10.1186/s42523-023-00241-z)
Supplement: Supplementary file 12 — Additional file 12: Ordination of the gut microbial communities. [file 42523_2023_241_MOESM12_ESM.docx]

# **Supplementary file 8.** A linear mixed effects model investigating the association between alpha diversity (Shannon Diversity Index and Chao1 Richness) and final brood size.

**
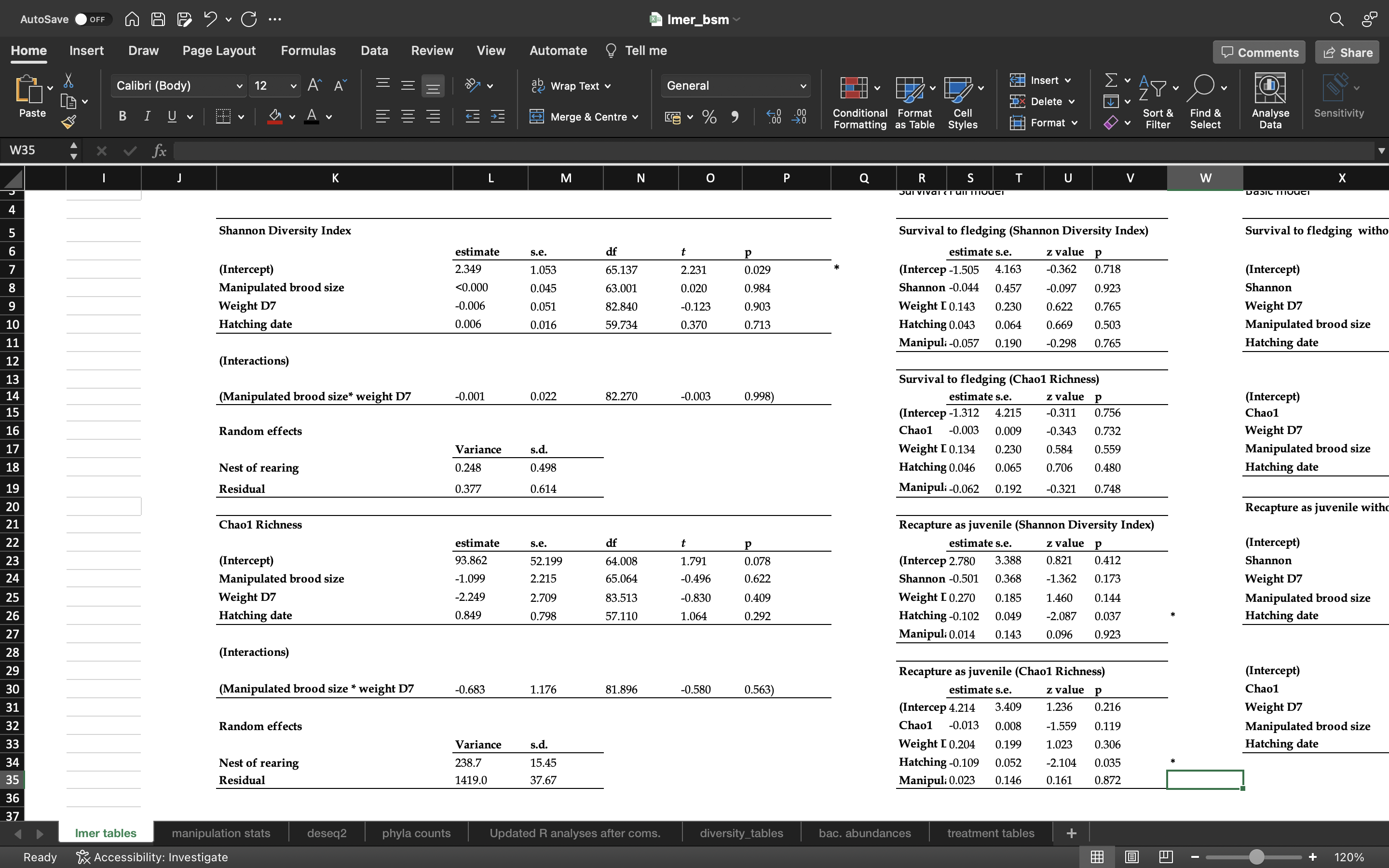
**

The model includes all four treatment groups i.e., the full model: control (C), unmanipulated control (COU), enlarged (E), and reduced (R). Interactions between alpha diversity and final brood size were removed as there was no significant interaction and are shown in the table below. Nest of rearing was included as a random effect to control for the non-independency of samples.
