# Supplementary material for: No evidence for associations between brood size, gut microbiome diversity and survival in great tit (Parus major) nestlings
Source: Anim Microbiome. 2023 Mar 22;5:19. doi: 10.1186/s42523-023-00241-z (PMC10031902; doi:10.1186/s42523-023-00241-z)
Supplement: Supplementary file 13 — Additional file 13: Differential analysis of abundance (DESeq2) to assess the ASV abundance between the treatment groups [file 42523_2023_241_MOESM13_ESM.docx]

# **Supplementary file 9.** A generalized linear model exploration into alpha diversity’s (Shannon Diversity Index and Chao1 Richness) association with short-term (survival to fledging) and mid-term (apparent juvenile) survival.

**
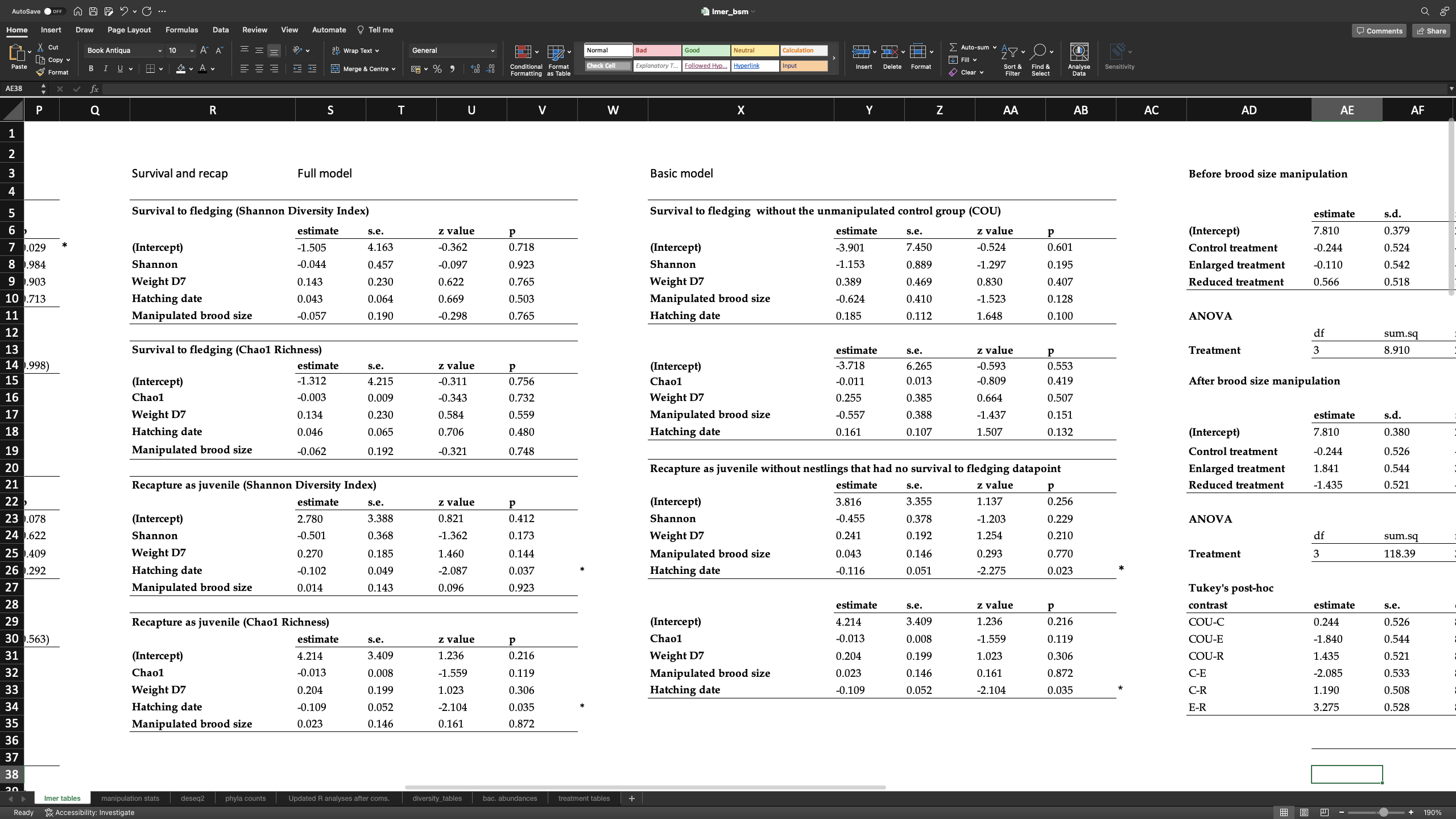
**

*Random effects were excluded as the model failed to converge.
